# Treatment-emergent adverse events after infusion of adherent stem cells: the MiSOT-I score for solid organ transplantation

**DOI:** 10.1186/1745-6215-13-211

**Published:** 2012-11-15

**Authors:** Johannes Dillmann, Felix C Popp, Barbara Fillenberg, Florian Zeman, Elke Eggenhofer, Stefan Farkas, Marcus N Scherer, Michael Koller, Edward K Geissler, Robert Deans, Deborah Ladenheim, Martin Loss, Hans J Schlitt, Marc H Dahlke

**Affiliations:** 1 Department of Surgery, University Hospital Regensburg, Franz Josef Strauss Allee 11, 93053, Regensburg, Germany; 2 Center for Clinical Studies, University Hospital Regensburg, Regensburg, Germany; 3 Athersys Inc., Cleveland, OH, USA

**Keywords:** Adherent adult stem cells, Mesenchymal stem cells, Multipotent adult progenitor cells, Solid organ transplantation, Immunotherapy, Scoring adverse events, Phase I trial

## Abstract

**Background:**

Cellular therapy after organ transplantation is emerging as an intriguing strategy to achieve dose reduction of classical immunosuppressive pharmacotherapy. Here, we introduce a new scoring system to assess treatment-emergent adverse events (TEAEs) of adherent stem cell therapies in the clinical setting of allogeneic liver transplantation (for example, the MiSOT-I trial Eudract CT: 2009-017795-25).

**Methods:**

The score consists of three independent modalities (set of parameters) that focus on clinically relevant events early after intravenous or intraportal stem cell infusion: pulmonary toxicity, intraportal-infusional toxicity and systemic toxicity. For each modality, values between 0 (no TEAE) and 3 (severe TEAE) were defined. The score was validated retrospectively on a cohort of *n*=187 recipients of liver allografts not receiving investigational cell therapy between July 2004 and December 2010. These patients represent a control population for further trials. Score values were calculated for days 1, 4, and 10 after liver transplantation.

**Results:**

Grade 3 events were most commonly related to the pulmonary system (3.5% of study cohort on day 4). Almost no systemic-related TEAEs were observed during the study period. The relative frequency of grade 3 events never exceeded 5% over all modalities and time points. A subgroup analysis for grade 3 patients provided no descriptors associated with severe TEAEs.

**Conclusion:**

The MiSOT-I score provides an assessment tool to score specific adverse events that may occur after adherent stem cell therapy in the clinical setting of organ transplantation and is thus a helpful tool to conduct a safety study.

## Background

The results of solid organ transplantation as definitive treatment for end-stage disease of the liver (for example, cirrhosis and metabolic decompensation) and other organs are clinically satisfactory [1]. However, the overall success of organ transplantation as a curative therapy is still hampered by the need for life-long immunosuppressive treatment of the recipient to control graft rejection. Standard-of-care immunosuppressive pharmacotherapy has a variety of drug-specific unwanted effects, such as the neurotoxicity of tacrolimus or the renal toxicity of ciclosporin [2]. Moreover, immunosuppressants increase the recipient’s risk of cancer [3] and opportunistic infections [4]. Immunomodulatory cellular therapy as an adjunct to classical pharmacotherapy has emerged as an intriguing strategy to achieve dose reductions of immunosuppressive drug therapy.

Multipotent adult progenitor cells (MAPCs) are bone marrow derived [5], adherent stem cells which are closely related to mesenchymal stem cells (MSC) [6], and have been shown to have immunosuppressive functions *in vitro* and *in vivo*[7]. MAPCs and MSCs effectively prolong allograft survival in small animal models when combined with otherwise subtherapeutic doses of suitable immunosuppressants, such as mycophenolate [8,9]. Building on this body of preclinical evidence, we have initiated a phase I study (MiSOT-I study, Eudract CT no. 2009-017795-25) to apply MAPCs after allogeneic liver transplantation (LTx) [10]. The primary endpoints of the MiSOT-I study will be safety and feasibility of MAPC infusions.

In the current paper, we introduce a scoring system designed to evaluate treatment-emergent adverse events (TEAEs) of intravenous and intraportal infusions of MAPCs after liver transplantation. Since similar events (mimicking ‘toxicity’) can also occur without cell therapy in LTx recipients, we validated the score in 187 recipients of liver allografts not receiving investigational cell therapy. We only focused on events that we anticipate to be specific for adherent stem cell therapy in this clinical setting. Hence, the current analysis outlines the background against which the toxicity of new cellular therapies has to be evaluated. The future objective of cell therapy after solid organ transplantation will be to establish the immunological efficacy of the cell product. Therefore, we also used the current analysis to establish a retrospective control group that will allow us to collect first evidence of the immunological efficacy of MAPC therapy after LTx.

## Methods

### Patients

**Figure 1:**
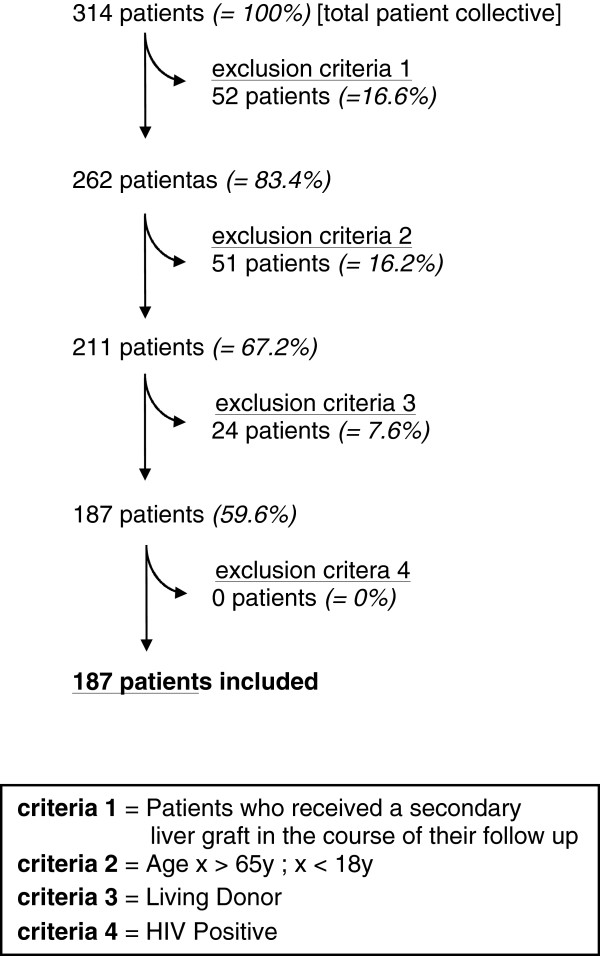
**Study population and exclusion criteria.***n*=187 patients are included out of an initial patient collective of *n*=314.

One hundred and eighty-seven patients who had received an allogeneic liver graft in our tertiary referral center between July 2004 and December 2010 were included in this retrospective analysis. Recipients of living-related grafts, patients who received a secondary liver graft during the entire course of their disease, HIV-positive recipients, and patients older than 65 years or younger than 18 years were excluded from the analysis (Figure 1). We have obtained ethical approval for this retrospective analysis from the local ethics committee (Ethikkommission der Universität Regensburg, No. 10-101-0244). Since patients data were analyzed in a pseudonymous fashion only, no informed consent was requested.

### Data collection

**Figure 2:**
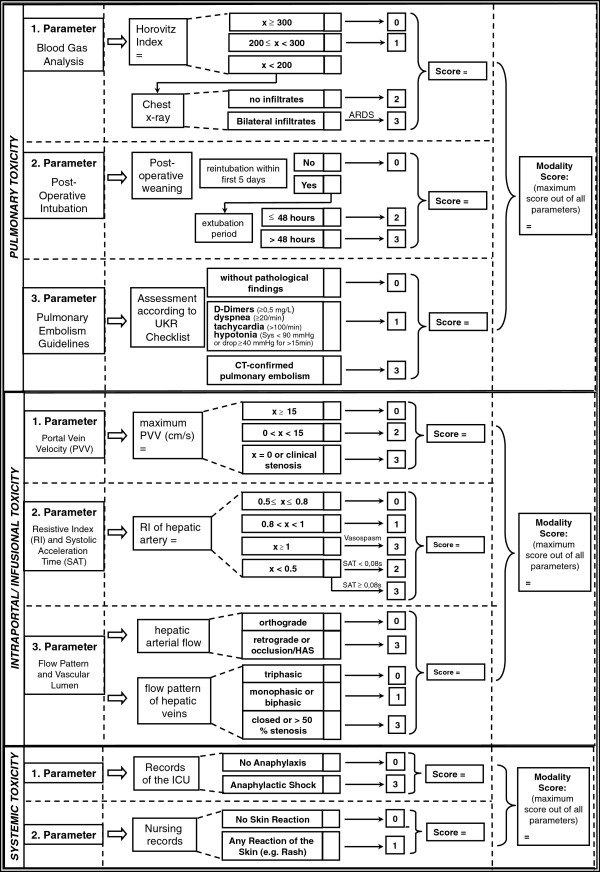
**Outline of the MiSOT-I score.** Every modality is defined by a set of parameters. Each parameter amounts to the designated score. The score values are not cumulative. Thus the maximum score in a set of parameters defines the score of that modality. ARDS, Acute respiratory distress syndrome; ICU, Intensive care unit; PVV, Portal vein velocity; RI, Resistive index; SAT, Systolic acceleration time; UKR, University Hospital Regensburg.

All patients included were reviewed using a standardized score sheet that will also be part of the MiSOT-I case report form (Figure 2). Clinical information from the Eurotransplant online database was also included. Additionally, the time to the last rejection-free follow-up was recorded as an indicator for the efficacy of standard-of-care immunosuppression. All data collected were stored and computed using IBM SPSS 18.0 Statistics Software (SPSS, Chicago, IL, USA). Data consistency was checked by a secondary investigator (BF, MHD).

### Statistical methods

Data analysis was carried out by a biostatistician (FZ). Proportions are presented as frequency counts and percentages, along with the corresponding 95% confidence intervals (CI) following Wilson’s method [11]. Continuous data are summarized as mean values and standard deviations. For comparison of two groups, Pearson’s chi-square test was applied for categorical variables and the Mann–Whitney *U*-Test for continuous variables. Rejection-free survival times were estimated by Kaplan-Meier analysis, and distributions between groups were compared by the log-rank test. All reported *P* values are two-sided, and a *P* value of 0.05 was considered the threshold of statistical significance. Hazard ratios (HR) and corresponding 95% CI were calculated and considered statistically significant if the CI excluded 1.0. No adjustment for multiple testing was performed.

### MiSOT-I score

The MiSOT-I score was designed as a high-barrier score that excludes clinical events that are clearly unacceptable TEAEs in phase I/II development of adherent stem cell products in patients after liver transplantation. Based on preclinical studies with adherent stem cell therapy [12,13] and observations made in a variety of early trials with adherent stem cells in indications other than solid organ transplantation [14-16], we defined three independent modalities (set of parameters) to reflect potentially critical aspects of adherent stem cell therapy: pulmonary toxicity (first-pass after intravenous injection), intraportal/infusional toxicity (first-pass after intraportal infusion), and systemic toxicity (immune reaction after cell infusion).

For each of the parameters of the MiSOT-I score, values between 0 and 3 were defined (Figure 2). A score of 0 implies no TEAE. Scores of 1 and 2 stand for intermediate TEAEs, while a score of 3 indicates a clinically unacceptable severe TEAE. In the prospective analysis of the MiSOT-I trial, a score of 3 will be considered a dose-limiting toxicity event.

Score values within a set of parameters were not cumulated so that the maximum score in each set of parameters defined the total score for that modality. Thus, each patient received three independent scores. To assess the clinical course of each patient, toxicity scores were computed for day 1 (range of days, 0 to 1), day 4 (range of days, 2 to 6), and day 10 (range of days, 8 to 12).

### Pulmonary toxicity

Assessment of pulmonary toxicity was based on three parameters, that is, the Horovitz Quotient (HQ) (FiO_2_/PaO_2_), the postoperative weaning from mechanical ventilation, and pulmonary embolism.

A HQ above 300 was defined as a score of 0, a HQ between 200 and 300 corresponded to a score of 1, and a HQ below 200 triggered further assessment of a chest X-ray for pulmonary infiltrations. Bilateral infiltrates as assessed by a staff radiologist corresponded to a score of 3 (equivalent to an acute respiratory distress syndrome), whereas the absence of such resulted in a score of 2 (equivalent to an acute lung injury) [17].

The course of postoperative weaning from mechanical ventilation was assessed as follows: Successful extubation without the need for reintubation within the first 48 h was assigned a score of 0 [18,19]. Reintubation within 48 h after extubation was assigned a score of 2, and reintubation more than 48 h after extubation within the first 5 postoperative days was assigned a score of 3.

The occurrence of CT-proven pulmonary emboli was assessed in accordance with European consensus guidelines [20]. A positive finding was defined as a score of 3, whereas the constellation of elevated D-Dimers (≥0.5 mg/L), dyspnea (tachypnea ≥20/min), tachycardia (>100/min), and hypotonia (systolic blood pressure <90 mmHg or a pressure drop of ≥40 mmHg for >15 min) was assigned a score of 1.

### Intraportal/infusional toxicity

The assessment of intraportal toxicity was based on hepatic duplex ultrasound results. The included parameters were as follows: the maximum portal venous velocity (PVV), the resistance index (RI) and systolic acceleration time (SAT) of the hepatic artery, and finally the flow pattern and patency of the hepatic vessels.

If the PVV was ≥15 cm/s a score of 0 was assigned. A PVV between 0 cm/s and 15 cm/s resulted in a 2, whereas a score of 3 was allocated in the case of portal venous occlusion (if a surgical problem was excluded: PVV = 0 cm/s, coherent post-stenotic flow acceleration, and clinical judgment) [21,22].

If the RI ranged between 0.5 and 0.8 a score of 0 was assigned. An RI above 0.8 but below 1 was given a score of 1. A RI <0.5 together with a SAT below 0.08 s resulted in a 2, whereas a score of 3 was allocated in the case of hepatic arterial occlusion (if a surgical problem was excluded: RI <0.5 together with a SAT ≥0.08 s [23], vasospasm indicated by a RI ≥1 [24], and clinical judgment).

Orthograde arterial blood flow and an open triphasic flow pattern of the hepatic veins were given a score of 0. Limited blood flow in the hepatic veins (monophasic or biphasic flow pattern without surgical impairment) [25] was assigned a score of 1. A score of 3 was allocated in the case of retrograde arterial blood flow, or an occlusion of the hepatic veins (>50% stenosis without a surgical problem). Although Doppler analysis is prone to inter- and intra-observer error, it was considered the best available tool for the assessment of hepatic perfusion [26].

### Systemic toxicity

The assessment of systemic toxicity was based on intensive care unit and nursing records. Any clinical finding implying an anaphylactic reaction was assigned a score of 3. Shock was defined by the need for vasopressor treatment or mechanical ventilation [27]. Any skin reaction was assigned a score of 1. The absence of anaphylaxis or skin reactions corresponded to a score of 0. A score value of 2 was not defined for this modality.

### Rejection analysis

The future objective of cell therapy after solid organ transplantation will be to establish the immunological efficacy of the cell product. Therefore, we also used the current analysis to determine the liver graft rejection-free survival of the patients in our study cohort after standard-of-care immunosuppressive treatment. These data will serve as a retrospective control group allowing us to collect any first evidence of the immunological efficacy of our cell therapy protocol.

We compared the rejection-rates of patients who received calcineurin inhibitors (ciclosporin A or tacrolimus) or sirolimus as their primary immusuppressive regimen (group CNI) to the patients treated with CNI-free immunosuppression (group CNI-free). We only retrospectively analyzed primary immunosuppression after liver transplantation. Therefore, any secondary changes to the immunosuppressive regimens were not taken into account. Rejection-free survival time of liver grafts was calculated from the date of graft implantation to the date of acute graft rejection. We differentiated between the following observation periods: day 0 to 10; day 0 to 30; day 0 to 90; day 0 to 365. Patients lost to follow-up and patients who died during the observation period were censored. For the primary rejection analysis, only biopsy-proven acute graft rejections or death from acute rejection were considered events. In a secondary rejection analysis, cortisone pulse therapy during the period on the intensive care unit was additionally considered an event for acute graft rejection.

## Results

### Study cohort

A total of 314 patients from our institutional database were included in this analysis (Figure 1). Patients who required a secondary liver graft in the course of their clinical follow-up were excluded from the study (52/314; 16.6%), as were children below the age of 18 years or patients older than 65 years (51/314; 16.2%), and patients who received a liver graft from a living donor (24/314; 7.6%). A HIV-positive status was also considered an exclusion criterion, however there were no HIV-positive patients among the initial 314 patients. Ultimately, 187/314 (59.6%) patients were included in the analysis; 72.7% of the study population were men. The mean age was 50.6 years (Table 1).

**Table 1:** **Baseline patient characteristics (*n*=187)**

		***n***	**%**	
*Sex*				
Male		136	72.7	
Female		51	27.3	
	**Mean**	**SD**	**Minimum**	**Maximum**
*Age (years)*	50.64	9.78	19.75	64.92

Gender and age of the patient collective included (*n*=187).

### Modality analysis

All patients were reviewed using a standardized score sheet (Figure 2). For the assessment of pulmonary toxicity, data for 187/187 patients (100%) were available on day 1, 85/187 (45%) on day 4, and 41/187 (21.9%) on day 10. For intraportal/infusional toxicity, data were retrieved for 152/187 patients (81.3%) on day 1, 113/187 (60.4%) on day 4, and 77/187 (41.2%) on day 10. Finally, for systemic toxicity, data were available for all patients on days 1, 4, and 10. Table 2 shows the score distribution for each of the three modalities on days 1, 4, and 10. At all time points examined, the majority of analyzed patients revealed no TEAEs. At no time did the frequency of severe TEAEs (grade 3) exceed 5%.

**Table 2:** Score distribution

**Modality**	**Score**	**Day 1**			**Day 4**			**Day 10**		
		***n***	**%**	**(95% CI)**	***n***	**%**	**(95% CI)**	***n***	**%**	**(95% CI)**
Pulmonary	Total *n*	187			85			41		
	0	91	65.9	(57.7-73.3)	47	55.3	(44.7-65.4)	22	53.7	(38.7-67.9)
	1	31	22.5	(16.3-30.1)	20	23.5	(15.8-33.6)	15	36.6	(23.6-51.9)
	2	12	8.7	(5.0-14.6)	15	17.6	(11.0-27.1)	4	9.8	(3.9-22.5)
	3	4	2.9	(1.1-7.21)	3	3.5	(1.2-9.9)	0	0.0	(0.0-8.6)
Intraportal/Infusional	Total *n*	152			113			77		
	0	98	64.5	(56.6-71.6)	82	72.6	(63.7-79.9)	59	76.6	(66.0-84.7)
	1	42	27.6	(21.1-35.2)	25	22.1	(15.5-30.6)	10	13.0	(7.2-22.3)
	2	9	5.9	(3.1-10.9)	6	5.3	(2.5-11.1)	7	9.1	(4.5-17.6)
	3	3	2.0	(0.07-5.6)	0	0.0	(0.0-3.3)	1	1.3	(0.2-7.0)
Systemic	Total *n*	187			187			187		
	0	186	99.5	(97.0-99.9)	185	98.9	(96.2-99.7)	186	99.5	(97.0-99.9)
	1	0	0.0	(0.0-2.0)	2	1.1	(0.29-3.82)	1	0.5	(0.09-2.97)
	3	1	0.5	(0.09-2.97)	0	0.0	(0.0-2.0)	0	0.0	(0.0-2.0)

Relative frequency of each score value within the three independent modalities.

CI, Confidence interval.

**Figure 3:**
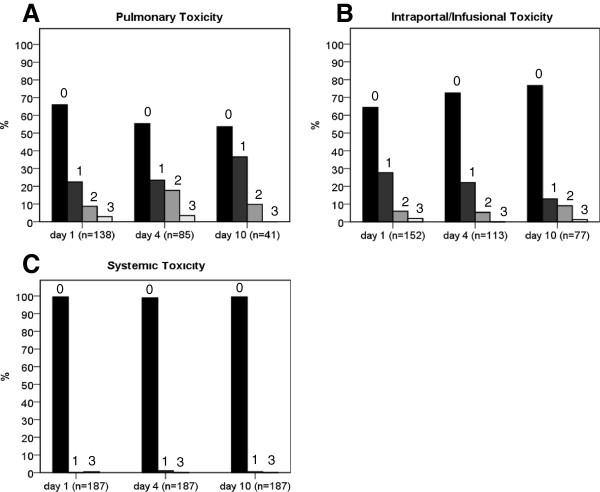
**Score distribution.** The relative frequency of each score value within the three independent modalities at days 1, 4, and 10. The value above each bar indicates the respective score value.

Figure 3A illustrates the distribution of pulmonary events. The highest relative frequency of grade 1 TEAEs (15/41; 36.6%) was observed on day 10, whereas grade 2 and 3 TEAEs were most frequently seen on day 4 (15/85; 17.6% and 3/85; 3.5%, respectively). The distribution of intraportal/infusional events is shown in Figure 3B. Grade 1 and 3 TEAEs were most frequent on day 1 (42/152; 27.6% and 3/152; 2%, respectively), while grade 2 TEAEs occurred most often on day 10 (7/77; 9.1%). Finally, Figure 3C outlines the distribution of systemic events. Grade 1 TEAEs occurred in 1.1% of patients (2/187) on day 4 and 0.5% of patients (1/187) on day 10. A grade 3 event occurred on only one occasion, that is, on day 1 in one patient (0.5%). All of the remaining patients revealed no systemic-related TEAEs (186/187; 99.5% on days 1 and 10, 185/187; 98.9% on day 4).

### Subgroup analysis for patients with grade 3 events

We further analyzed the subgroup of 12 patients who developed clinically unacceptable severe TEAEs (grade 3 events) to identify clinical conditions typically associated with such events. None of the 12 patients attained a score of 3 in more than one modality. Moreover, no patient experienced grade 3 events on 2 different days within a single modality. The following clinical events were responsible for grade 3 TEAEs: one patient experienced a pulmonary embolism on day 1; two patients were reintubated within the first 5 postoperative days after an extubation period >48 h; three patients developed acute respiratory distress syndrome on day 1 and one on day 4; one patient had a portal venous occlusion on day 1; two patients experienced a hepatic arterial occlusion on day 1; one patient suffered an occlusion of the hepatic veins on day 10, and one patient had an anaphylactic shock on day 1. To determine which patient characteristics are associated with severe TEAEs and to develop hypotheses for the early detection of these patients, the group of 12 patients with grade 3 TEAEs was compared with the remaining 175 patients. However, none of the parameters analyzed revealed a significant difference between the two groups (Table 3).

**Table 3:** Score 3 risk factor profile

***n***	**Parameter**	***n***	**Score ≠ 3**	***n***	**Score = 3**	***P***
187	Gender (% male)	175	71.4	12	91.7	0.128
187	Age (years)	175	50.4 (SD 9.9)	12	53.8 (SD 8.4)	0.328
175	Cold ischemic period (Hours:Minutes)	164	10:09 (SD 2:19)	11	10:13 (SD 2:08)	0.417
183	Immunosuppression (% including CNI)	171	65,7	12	83.33	0.342
187	Blood type (% Type A)	175	45.7	12	58.3	0.397
187	Blood type (% Type B)	175	10.3	12	8.3	0.829
187	Blood type (% Type O)	175	35.4	12	33.3	0.883
187	Alcoholic liver cirrhosis as cause for LTx (%)	175	36.6	12	33.3	0.822
145	Last measured Gamma GT of donor (U/L)	137	55 (SD 54.7)	8	73.75 (SD 58.2)	0.343
148	Last measured Bilirubin (total) of donor (μmol/L)	140	9.59 (SD 11.3)	8	20.24 (SD 22.7)	0.211
135	Last measured alcalic phosphatase of donor (IU/L)	127	72.52 (SD 40.6)	8	73.63 (SD 27.6)	0.734

Statistical analysis of potential parameters correlating with the group of patients, who attained a score of 3 in one of the modalities.

SD, Standard deviation.

### Rejection-free survival

For the assessment of the liver graft rejection-free survival, data were available for 185/187 patients (98.9%). One patient died prior to first immunosuppressive treatment. For the other patient, it was not possible to accurately determine the primary immunosuppression after LTx retrospectively. As secondary changes to the immunosuppressive regimen and special patient characteristics (for example, renal failure, high MELD scores) were not considered, the calculated rejection rates cannot be considered to have prospective impact. However, as a best available retrospective group, these data will be valuable to establish any first efficacy of MAPC therapy.

**Figure 4:**
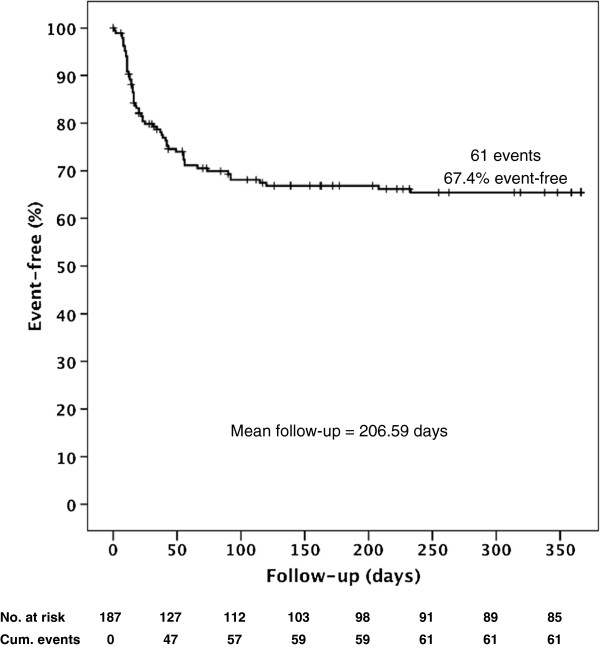
**Kaplan-Meier curve of rejection-free graft survival 365 days after liver transplantation patients with biopsy-proven acute graft rejection or patients who died due to an acute rejection were considered events.** Patients who died of other causes and patients lost to follow-up were censored.

Among the 129 patients of the CNI group, 94 (72.9%) did not reject their grafts (biopsy-proven) and were thus rejection-free during a follow-up period of 365 days. Fifty-six patients were treated with CNI-free immunosuppression. These patients were mainly patients with particularly high MELD scores and pronounced renal impairment. Thirty-two of these patients (57.1%) did not experience acute rejection during clinical follow-up (HR=1.96, 95%CI: (1.17; 3.30), *P*=0.01). The total rejection-free survival of all patients regardless of immunosuppressive treatment (also including the two dropouts) was 67.4% (126/187) (Figure 4). In the early postoperative phase until day 10, only 5/129 (3.9%) of the CNI-treated patients rejected their liver graft, compared to 5/56 (8.9%) patients in the CNI-free group (HR=2.41, 95%CI: (0.70; 8.32), *P*=0.17). A comparison on day 30 (12.4% *vs.* 33.9%: HR=3.09, 95%CI: (1.59; 6.01), *P* <0.01) and day 90 (22.4% *vs.* 42.9%: (HR=2.36, 95%CI: (1.37; 4.06), *P* <0.01) emphasized the difference between the two patient collectives.

Finally, a secondary retrospective rejection analysis was performed that also considered the application of a cortisone pulse therapy during the ICU period as an indicator of suspected and treated acute graft rejection. This analysis revealed an additional eight cases (six CNI *vs.* two CNI-free) of non-biopsy-proven acute graft rejection with the cortisone bolus as the only indicator of an acute liver graft rejection. All of these events occurred within the first 30 postoperative days, increasing the rejection rate for the CNI group from 12.4% (16/128) to 17.1% (22/129), and for the CNI-free group from 33.9% (19/56) to 37.5% (21/55).

## Discussion

The objective of this study was to retrospectively validate a newly designed scoring system for TEAEs of liver-directed adherent stem cell therapy after liver transplantation (Figure 2). This score will be used in an approved phase-I study (MiSOT-I). The validation was conducted retrospectively in a cohort of 187 recipients of liver allografts who did not receive investigational cell therapy.

The majority of the study population showed no TEAEs (score 0) in accordance with the MiSOT-I score. This was expected, because the cut-off values of the score parameters were chosen to be a high barrier for clearly unacceptable clinical events in the further development of this and other stem cell therapies. For all modalities, the relative frequency of severe TEAEs (score 3) did not exceed 5%. Previous studies looking at comparable complications after organ transplantation have shown rates of pulmonary embolism or acute respiratory distress syndrome of 0.37% [28] and 5.5% [29], respectively. Also, portal venous occlusion, hepatic artery thrombosis, and hepatic vein stenosis, which accounted for most of the intraportal/infusional grade 3 TEAEs in our cohort, have reported rates of up to 2.6%, 3.2%, and 1.5%, respectively [30,31].

By contrast, anaphylactic reactions (grade 3 parameter for systemic toxicity) are extremely rare in the clinical setting of solid organ transplantation, to our knowledge only one such case has been described in the published literature [32]. When comparing the three modalities, the highest relative frequency of a score of 3 was most often pulmonary-related (day 4 = 3.5%). This is consistent with previous studies suggesting a high rate of pulmonary complications following orthotopic liver transplantation [29]. Systemic TEAEs were the least frequent, which can be explained by the general low incidence of postoperative anaphylaxis [32]. Thus, in view of the grade 3 events in our cohort, the results of this study confirm and further quantify the findings in the literature concerning pulmonary, hepatic, and systemic function after deceased-donor liver transplantation.

A further subgroup analysis for patients with grade 3 events failed to provide a valuable hypothesis on which descriptors are associated with severe TEAEs (Table 3). Previous investigations have shown that patients with alcoholic cirrhosis achieve the same postoperative survival and complication rates as non-alcohol-related transplantations [33]. However, for all remaining parameters, numerous studies show that high age [34], male gender [35], non-A blood type of the recipient [35], low donor creatinine or bilirubine [36], a long cold ischemia time [37], and a high MELD score [38] all significantly correlate with an increased postoperative morbidity and mortality rate after liver transplantation. Hence, a correlation between grade 3 TEAEs and any of these parameters was expected but was not established in our cohort. A possible explanation for this discrepancy is the difference in size of the two compared subgroups (*n*=12 *vs. n*=175), although this was considered in the design of the statistical analysis. Another reason for this observation may be the choice of exclusion criteria (Figure 1), since most previous comparative studies included re-transplanted patients and patients above 65 years of age.

Biopsy-proven rejection-free survival after non-living related orthotopic liver transplantation was analyzed. Here, we grouped patients receiving CNI-free, sirolimus-free, bottom-up immunosuppression (as will be used in the MISOT-I study) against all other patients [39]. Patients who received ciclosporin A, tacrolimus, or sirolimus as their primary immunosuppression presented with a rejection-free graft survival rate of 72.9% after 365 days of follow-up (mean follow-up = 206.59 days). The non-CNI, non-sirolimus, bottom-up group presented with rejection-free survival of 57.1% in comparison. Since time to biopsy-proven acute rejection will be a secondary endpoint of the MiSOT-I trial, the analysis of the rejection time in the present cohort can serve as a retrospective comparator, naturally with all bias and shortcomings of such an analysis.

Unavailability of data and low data consistency over the analyzed time period was the key limitation of this retrospective study. However, in the light of no other available data, this study will still be the most valuable comparator for MiSOT-I and other investigational phase I studies applying adherent stem cell therapies. No high-risk patients for the elected events could be identified from our present cohort and, therefore, we have no further means to exclude patient groups from MiSOT-I.

## Conclusion

Whether adherent stem cell therapy is indeed inherently safe for all patients remains to be determined. In any case, the current score appears suitable to identify problems of adherent stem cell infusions, at least in the areas that we have included. Since the frequency of grade 3 TEAEs in this retrospective analysis was never higher than 5%, we may assume a probability of <5% that complications that are identified by this score are related to standard-of-care treatment after liver transplantation. Consequently, in the clinical setting of the MiSOT-I study, in which we plan to administer MAPCs to patients after liver transplantation, the probability of a single event being stem-cell-related is greater than 95% and the probability of two consecutive events being stem-cell-related is greater than 99.75%. Therefore, two grade 3 events have been defined as a stopping rule for MiSOT-I.

## Competing interests

The authors declare no competing interests.

## Authors’ contributions

JD and MHD designed the primary outlines of the MiSOT-I score with EE, FCP, ML, SF, MNS, and HJS. BF provided essential documents to realize this study. FZ and MK contributed substantially by ensuring statistical accuracy. FCP, EKG, HJS, RD, and DL supported the study with their knowledge and experience. All authors have read and approved the final manuscript.
